# Midazolam and propofol used alone or sequentially for long-term sedation in critically ill, mechanically ventilated patients: a prospective, randomized study

**DOI:** 10.1186/cc13922

**Published:** 2014-06-16

**Authors:** Yongfang Zhou, Xiaodong Jin, Yan Kang, Guopeng Liang, Tingting Liu, Ni Deng

**Affiliations:** 1 Department of Critical Care Medicine, West China Hospital of Sichuan University, Chengdu 610041, China

## Abstract

**Introduction:**

Midazolam and propofol used alone for long-term sedation are associated with adverse effects. Sequential use may reduce the adverse effects, and lead to faster recovery, earlier extubation and lower costs. This study evaluates the effects, safety, and cost of midazolam, propofol, and their sequential use for long-term sedation in critically ill mechanically ventilated patients.

**Methods:**

A total of 135 patients who required mechanical ventilation for >3 days were randomly assigned to receive midazolam (group M), propofol (group P), or sequential use of both (group M-P). In group M-P, midazolam was switched to propofol until the patients passed the spontaneous breathing trial (SBT) safety screen. The primary endpoints included recovery time, extubation time and mechanical ventilation time. The secondary endpoints were pharmaceutical cost, total cost of ICU stay, and recollection to mechanical ventilation-related events.

**Results:**

The incidence of agitation following cessation of sedation in group M-P was lower than group M (19.4% versus 48.7%, *P* = 0.01). The mean percentage of adequate sedation and duration of sedation were similar in the three groups. The recovery time, extubation time and mechanical ventilation time of group M were 58.0 (interquartile range (IQR), 39.0) hours, 45.0 (IQR, 24.5) hours, and 192.0 (IQR, 124.0) hours, respectively; these were significantly longer than the other groups, while they were similar between the other two groups. In the treatment-received analysis, ICU duration was longer in group M than group M-P (*P* = 0.016). Using an intention-to-treat analysis and a treatment-received analysis, respectively, the pharmaceutical cost of group M-P was lower than group P (*P* <0.01) and its ICU cost was lower than group M (*P* <0.01; *P* = 0.015). The proportion of group M-P with unbearable memory of the uncomfortable events was lower than in group M (11.7% versus 25.0%, *P* <0.01), while the proportion with no memory was similar (*P* >0.05). The incidence of hypotension in group M-P was lower than group (P = 0.01).

**Conclusion:**

Sequential use of midazolam and propofol was a safe and effective sedation protocol, with higher clinical effectiveness and better cost-benefit ratio than midazolam or propofol used alone, for long-term sedation of critically ill mechanically ventilated patients.

**Trial registration:**

Current Controlled Trials ISRCTN01173443. Registered 25 February 2014.

## Introduction

Critically ill, mechanically ventilated patients receive sedation to eliminate or relieve their anxiety and discomfort; to facilitate specific procedures such as tracheal suctioning and frequent venipuncture; to reduce oxygen consumption; and to decrease complications such as unplanned extubation. An ideal sedative should have a rapid onset, a short duration of action, a lack of accumulation, ease of titration and administration, and no cardiovascular and respiratory depression [1,2]. Until now, clinicians have been struggling to identify the ideal drug candidates to treat critically ill patients.

Recent surveys showed that midazolam and propofol remain the dominant medications used for ICU sedation [3-5]. The two drugs are equally safe and effective for short-term sedation [6,7]. However, each drug is associated with adverse effects when used for long-term sedation. Treatment with midazolam may cause acute withdrawal syndrome and delayed recovery from drug accumulation, especially in patients with chronic renal failure [6-13]. Propofol treatment causes dose-dependent effects and faster recovery with no accumulation [6,7,12,13]. However, propofol may cause hypertriglyceridemia and cardiovascular depression, and is associated with the risk of propofol infusion syndrome and high pharmaceutical cost [7,8,11,13-16]. In view of the limitations associated with these drugs when used alone, our study evaluated whether the sequential use of midazolam and propofol in the long-term sedation of critically ill, mechanically ventilated patients, reduced the adverse effects.

## Materials and methods

### Patients

We conducted a single-center, randomized, open-label trial in West China Hospital of Sichuan University, Sichuan, China during March 2010 to September 2011. The study was approved by the ethics committee of West China Hospital of Sichuan University in accordance with the Helsinki Declaration. Written informed consent was obtained from the patients’ authorized surrogates. Critically ill patients (*n* = 135) undergoing mechanical ventilation were enrolled in this study within 1 hour of admission to the ICU. Patients transferred to the 132-bed multidisciplinary closed ICU from the medical, surgical, and other departments (intubated) were considered for the study.

Patients were selected based on the following inclusion criteria: intubated patients (>18 years old) who were expected to receive long-term (≥72 hours) mechanical ventilation on admission to the ICU. The exclusion criteria of the study were as follows: known or suspected allergy to propofol or midazolam, suspected pregnancy, gross obesity, hyperlipemia, moribund state, history of alcoholism or intake of anti-anxiety drugs or hypnotics, chronic renal failure, coma by cranial trauma or neurosurgery or unknown etiology or status epilepticus, and unwillingness to provide informed consent by patients or their authorized surrogates following ICU admission.

### Study procedures

#### Randomization

The eligible patients were randomly assigned in a 1:1:1 manner to receive midazolam, propofol, or sequential use of midazolam and propofol. Each assignment was concealed by random selection of opaque, sealed envelopes for consecutive patients from a box of 135 envelopes. Every envelope contained a number by a random allocation process using a computer-generated random block design.

### Treatment interventions

All patients received continuous intravenous fentanyl for analgesia with a bolus dosage of 1 to 2 μg/kg and a maintenance dosage of 1 to 2 μg/kg/hour. Patients allocated to the midazolam group (group M) were treated with an infusion bolus of 0.03 to 0.30 mg/kg and continuous infusion of 0.04 to 0.20 mg/kg/hour, with the dosage adjusted to achieve the desired level of sedation. Patients allocated to the propofol group (group P) received an infusion bolus of 0.50 to 3.00 mg/kg and continuous infusion of 0.50 to 3.00 mg/kg/hour, with the dosage adjusted to achieve the desired level of sedation. Patients allocated to the sequential use of midazolam and propofol group (group M-P) first received midazolam by the same method as group M. When patients met the sequential criteria, midazolam was switched to propofol, which was administered at the maintenance dosage of 0.50 to 3.00 mg/kg/hour. Patients passed the spontaneous breathing trial (SBT) safety screen when they showed adequate oxygenation (oxygen partial pressure ≥60 mmHg, fraction of inspired oxygen ≤50%, and positive end-expiratory pressure ≤10 cmH_2_O), hemodynamic stability with no evidence of myocardial ischemia and hypotension, and no significant use of vasopressors (dopamine or dobutamine ≤5 μg/kg/minute or norepinephrine ≤2 μg/minute) [17].

The Riker sedation–agitation scale (SAS) [18,19] was used to assess the sedation quality in each group. According to our local sedation procedure, the nurses continuously monitored the sedation depth and adjusted the dosages of sedative and analgesic drugs to maintain the sedation target level to 3 or 4 points of degree on the SAS scale. SAS scores were recorded every 4 hours (or more frequently when indicated) by the nursing staff to ensure correct titration of the sedative infusion.

Sedation quality was calculated as follows:

$$\begin{array}{ll} \mathrm{Sedation} & \mathrm{satisfaction}\;\mathrm{degree}=\mathrm{times}\;\mathrm{that}\;\mathrm{SAS}\;\mathrm{score}\;\mathrm{in}\;\mathrm{the}\\ & \;\mathrm{target}\;\mathrm{level}\;\mathrm{range}/\mathrm{total}\;\mathrm{evaluation}\;\mathrm{times}\times 100\%. \end{array}$$

Starting the next day after enrolment, the patients were assessed every morning by the physicians with a daily interruption of the continuous sedation safety screen [17] when there were no contraindications, including severe hypoxemia, myocardial ischemia, hypertensive crisis, status asthmaticus, sustained agitation with the increased use of sedation drugs, and treatment with neuromuscular blockers. Subsequently, the sedative and analgesic infusion was interrupted until the patients were awake, determined based on the following three simple tasks: opening their eyes, squeezing the hand and moving fingers, and expressing discomfort. When patients developed sustained agitation, marked dyspnea, pulse oxymetry <88% for ≥5 minutes or arrhythmias, sedatives were restarted at one-half of the previous dose and then titrated to achieve the target sedation level. The patients were reassessed the following morning.

### Weaning and extubation

Starting the next day after enrolment, the respiratory therapists managed patients with the SBT safety screen every morning. Patients in each group who passed the SBT safety screen underwent a 30-minute SBT with a pressure support ventilation of 8 cmH_2_O, positive end-expiratory pressure of 5 cmH_2_O, and fraction of inspired oxygen of 40% [20]. Patients failed the SBT trial if their respiratory rate was >35 breaths/minute or <8 breaths/minute; they showed hypoxemia (oxygen saturation <90%), abrupt changes in mental status, hemodynamic instability with heart rate and blood pressure changing more than 20% from the previous level, or acute cardiac arrhythmia, tachycardia (>140 beats/minute), or bradycardia (<60 beats/minute); they showed marked dyspnoea; or they showed use of accessory muscles or abdominal paradox. The ventilation support level was immediately restored for the patients who failed the SBT. Patients were reassessed the following morning. Patients passed the SBT if they did not exhibit any failure signs. When the SBT was successful, physicians and respiratory therapists decided to extubate the patients, including timing the discharge of the patients from the ICU.

### Outcome measurements

The primary outcome measures included the recovery and extubation time, which were defined as the time from the cessation of sedation until awakening and extubation, respectively. The data were also collected for the duration of sedation, duration of mechanical ventilation, length of stay in ICU and hospital, ICU mortality and hospital mortality, occurrence of hypotension (decrease in systolic blood pressure >20%) during the sedation period, presence or absence of agitation during the 4 hours after stopping sedation, serum triglyceride concentrations at admission and the stopping of sedation, and the incidence of acute kidney injury according to Acute Kidney Injury Network criteria during the study period [21].

The secondary endpoints were calculation of the cost of sedation, the primary monetary pharmaceutical costs of sedation and total ICU costs (including ICU therapy and sedation). After patients were transferred out of the ICU, their recollection to mechanical ventilation-related events (rolling over, suction, and endotracheal tube stimulation and pain) were recorded using a questionnaire. The patients responded from the following options: unbearable memory, bearable memory, and no memory.

### Statistical analysis

Statistical power was estimated using reduction in recovery time as the primary outcome. The mean (±standard deviation) recovery time for midazolam was 54.7 ± 12.3 hours in the long-term sedation group (that is, >7 days) according to Carrasco and colleagues [7]. A sample size of 105 (35 patients in each group) was thus considered adequate to detect a 15% relative reduction in recovery time for propofol or the sequential use of midazolam and propofol with 90% power and a two-sided significance level of 0.05. As some patients were severely ill or expected to abandon the treatment, 135 patients were enrolled for the study in order to manage the drop-outs.

Values of normal and non-normal distributions were expressed by mean ± standard deviation and median, respectively. The differences in normally distributed data of the three groups were compared with one-way analysis of variance. The data between any of the two groups were analyzed with Student–Newman–Keuls methods of analysis of variance. *P* < 0.05 was considered statistically significant. Non-normally distributed parameters were compared with Kruskal–Wallis analysis of variance, and in the presence of significant difference in the three groups (*P* < 0.05) the data between any of the two groups were analyzed with Mann–Whitney analysis of variance with the value of α adjusted to 0.017. Nominal data were analyzed by either the chi-squared test or Fisher's exact test. Statistically significant differences between any two groups were further compared with the value of α adjusted to 0.017.

## Results

A total of 135 patients were enrolled in the study. We excluded 11 patients because their condition deteriorated rapidly and they died in the 48 hours after admission. A total of 104 patients completed the study protocol, 11 patients withdrew (including three patients who were transferred to the Tibet Chengban branch of our hospital, and eight patients who were transferred to their local hospitals and two of these patients who died), seven patients had tracheotomy, and two patients showed therapeutic failure (due to inadequate sedation). In total, 124 patients were included in the intention-to-treat analysis: 43 patients were sedated with midazolam (group M), 42 patients sedated with propofol (group P), and 39 patients were treated with midazolam and propofol sequentially (group M-P). No differences were observed among the groups with respect to demographics (age, sex and weight) or baseline parameters of SAS score, Sequential Organ Failure Assessment score [22], and diagnosis for admission to the ICU (shown in Table 1).

**Table 1:** Baseline demographic data

**Variable**	**Group M (*n*** **= 43)**	**Group P (*n*** **= 42)**	**Group M-P (*n*** **= 39)**	***P *** **value**
Age (years)	54.8 ± 13.6	55.9 ± 18.0	53.2 ± 16.8	0.75
Weight (kg)	60.1 ± 10.2	58.4 ± 9.8	59.6 ± 9.2	0.71
Gender (male/female)	26/17	24/18	24/15	0.91
SOFA score at enrollment	7.9 ± 2.5	8.3 ± 2.0	9.0 ± 2.0	0.10
SAS score at enrollment	5.6 ± 0.9	5.5 ± 0.8	5.4 ± 0.8	0.77
Diagnosis at ICU admission				
Sepsis	6	6	5	1.00
COPD/CHF	3	4	3	0.92
Pneumonia	6	8	7	0.83
Pancreatitis	8	6	5	0.82
Trauma	14	10	12	0.65
Postoperative				
Vascular surgery	1	2	3	0.44
Liver transplant	1	1	0	1.00
Abdominal surgery	4	5	4	0.88

Values are expressed as mean ± standard deviation. CHF, congestive heart failure; COPD, chronic obstructive pulmonary disease; group M, midazolam group; group M-P, midazolam and propofol group; group P, propofol group; SAS, Riker sedation–agitation scale at ICU admission; SOFA, Sequential Organ Failure Assessment at ICU admission. Normally distributed data were compared using one-way analysis of variance. The Student–Newman–Keuls test was used for *post hoc* multiple comparisons. Nominal data comparisons were based on either the chi-square test or Fisher's exact test.

### Quality of sedation

**Figure 1:**
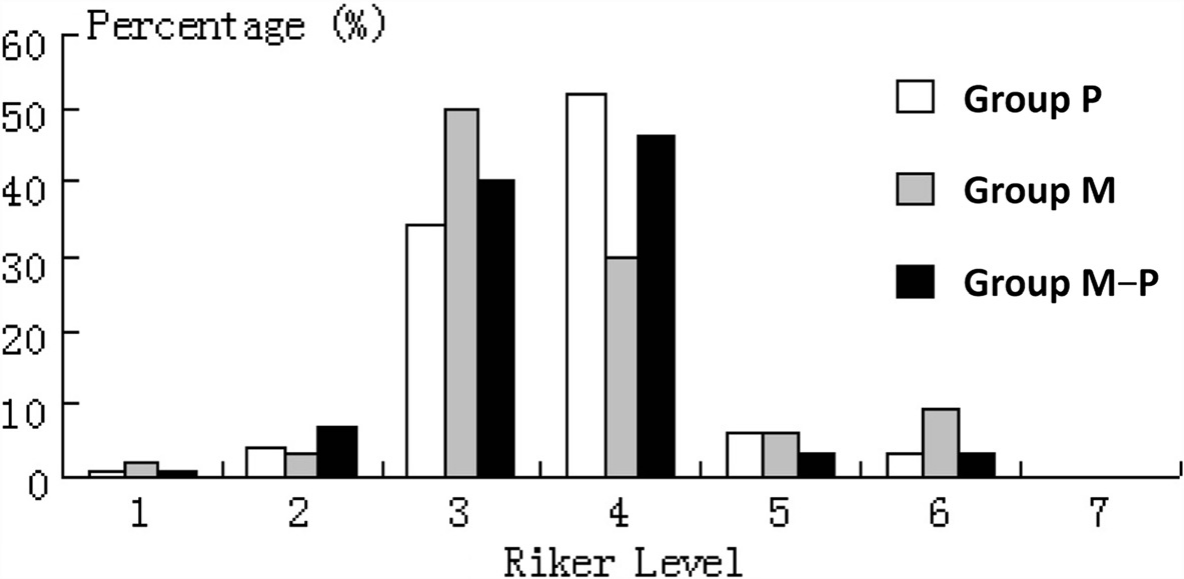
Percentage of total evaluation times of sedation reached for each degree of Riker sedation–agitation scale score with propofol, midazolam, and sequential use of midazolam and propofol.

The mean percentages for total times of adequate sedation in group M, group P and group M-P were 78%, 84% and 86%, respectively (*P* > 0.05). The percentage of agitated patients (13 patients were missing assessments) in group M (48.7%, 19/39) was higher than in group P (22.2%, 8/36; *P* = 0.03) and group M-P (19.4%, 7/36; *P* = 0.01). The percentage of total evaluation times of sedation reached for each degree of SAS scores with propofol, midazolam, and sequential use of midazolam and propofol are shown in Figure 1.

### Study outcomes

Data for the outcome variables determined using an intention-to-treat analysis are presented in Table 2. No difference was detected in the median doses of fentanyl among groups (*P* = 0.26): group M, 0.86 (interquartile range (IQR), 0.66) μg/kg/hour; group P, 0.92 (IQR, 0.13) μg/kg/hour; group M-P, 0.78 (IQR, 1.03) μg/kg/hour. No significant differences were observed in the duration of sedation and the length of stay in ICU and hospital among groups (*P* > 0.05); however, the midazolam sedation time in group M-P was shorter than in group M (*P* = 0.01). The recovery times in group M, group P and group M-P were 58.0 (IQR, 39.0) hours, 1.5 (IQR, 1.0) hours, and 1.0 (IQR, 1.0) hours, respectively. The extubation times were 45.0 (IQR, 24.5) hours, 3.0 (IQR, 1.0) hours, and 2.0 (IQR, 1.5) hours, respectively. The mechanical ventilation times were 192.0 (IQR,124.0) hours, 126.0 (IQR, 71.7) hours, and 114.8 (IQR, 95.5) hours, respectively, for the three groups. These values in group M were significantly longer than in the other two groups (*P* < 0.05), but no significant differences were seen between group P and group M-P (*P* > 0.05). No differences in ICU mortality, hospital mortality, incidence of acute kidney injury, or numbers of patients received continuous renal replacement therapy were detected among groups (*P* > 0.05).

**Table 2:** Study outcomes using intention-to-treat analysis

**Data**	**1 Group M (*n*** **= 43)**	**2 Group P (*n*** **= 42)**	**3 Group M-P (*n*** **= 39)**	***P *** **value**	***Post hoc *test (*P*** **< 0.017)**
Sedation time (hours)	142.0 (94.5)	120.0 (70.0)	112.5 (101.0)	0.47	
Midazolam sedation time (hours)^a^	142.0 (94.5)	–	77.0 (82.0)	<0.01	
Mechanical ventilation time (hours)	192.0 (124.0)	126.0 (71.7)	114.8 (95.5)	0.01	1 vs. 2; 1 vs. 3
Recovery time^b^ (hours)	58.0 (39.0)	1.5 (1.0)	1.0 (1.0)	<0.01	1 vs. 2; 1 vs. 3
Extubation time^c^ (hours)	45.0 (24.5)	3.0 (1.0)	2.0 (1.5)	<0.01	1 vs. 2; 1 vs. 3
ICU duration (days)	17.0 (7.5)	10.0 (16.0)	12.5 (8.0)	0.04	
Length of stay in hospital (days)	23.0 (26.5)	17.0 (40.0)	28.5 (31.5)	0.24	
Incidence of AKI	9 (20.9%)	8 (19.0%)	11 (28.2%)	0.59	
Numbers of patients receiving CRRT	4 (9.3%)	3 (7.1%)	5 (12.8%)	0.70	
ICU mortality	7 (16.3%)	6 (14.3%)	4 (10.3%)	0.76	
Hospitalization mortality	10 (23.3%)	7 (16.7%)	6 (15.4%)	0.68	

Data are expressed as median (interquartile range) for non-normally distributed data or number (%) for nominal data. Kruskal–Wallis analysis of variance was used for non-normally distributed data comparisons, and the nominal data comparisons were based on either the chi-squared test or Fisher's exact test. In the presence of a significant difference in the three groups (*P* < 0.05), the parameters between any two groups were further compared with the value of α adjusted to 0.017. AKI, acute kidney injury; CRRT, continuous renal replacement therapy. ^a^Medazolam sedation times of group M and group M-P were analyzed by independent two-sample *t* test. ^b^Time from the stopping of sedation until awake: missing assessments for the patients withdrawing from treatment or suffering from therapeutic failure (four patients in group M, six patients in group P, and three patients in group M-P). ^c^Time from the stopping of sedation until extubation: exposure calculation was based on patients completing treatment (including 37 patients in group M, 35 patients in group P, and 32 patients in group M-P), patients withdrawing treatment and suffering therapeutic failure, and those who underwent tracheostomy without determining the extubation.

Data for the study outcomes of the 104 patients completing this study protocol using treatment-received analysis are presented in Table 3. Patients of group P and group M-P were both associated with earlier extubation, faster recovery and shorter mechanical ventilation than those in group M (*P* < 0.05). The ICU duration in group M-P was shorter than in group M (*P* = 0.016). There were no differences in the duration of sedation, hospital length of stay, ICU mortality, or hospital mortality among groups (*P* > 0.05).

**Table 3:** **Analysis of treatment outcomes**^**a**^

**Data**	**1 Group M (*n*** **= 37)**	**2 Group P (*n*** **= 35)**	**3 Group M-P (*n*** **= 32)**	***P *** **value**	***Post hoc *test (*P*** **< 0.017)**
Sedation time (hours)	142.0 (94.5)	120.0 (70.0)	112.5 (101.0)	0.73	
Mechanical ventilation time (hours)	192.0 (124.0)	126.0 (71.7)	114.8 (95.5)	0.02	1 vs. 2; 1 vs. 3
Recovery time^b^ (hours)	45.0 (26.0)	1.5 (1.0)	1.0 (1.0)	<0.01	1 vs. 2; 1 vs. 3
Extubation time^c^ (hours)	45.0 (24.5)	3.0 (1.0)	2.0 (1.5)	<0.01	1 vs. 2; 1 vs. 3
Length of stay in hospital (days)	23.0 (26.5)	17.0 (40.0)	28.5 (31.5)	0.24	
ICU duration (days)	17.0 (7.5)	10.0 (16.0)	12.5 (8.0)	0.04	1 vs. 3
ICU mortality	5 (13.5%)	6 (17.1%)	4 (12.5%)	0.85	
Hospitalization mortality	7 (18.9%)	7 (20.0%)	4 (12.5%)	0.68	

Data expressed as median (interquartile range) for non-normally distributed data or number (%) for nominal data. Kruskal–Wallis analysis of variance was used for non-normally distributed data comparisons, and the nominal data comparisons were based on either the chi-squared test or Fisher's exact test. In the presence of a significant difference in the three groups (*P* < 0.05), the parameters between any two groups were further compared with the value of α adjusted to 0.017. ^a^Calculation of 104 patients in the per-treatment-received analysis. ^b^Time from the stopping of sedation until awake. ^c^Time from the stopping of sedation until extubation.

### Pharmaceutical sedation costs and total cost of treatment in the ICU

Non-normally distributed data pertaining to the pharmaceutical sedation costs and the total cost of ICU treatment are presented in Table 4. The median pharmaceutical costs of sedation in group M, group P, and group M-P were 1,982 (IQR, 2,348) yuan, 3,744 (IQR, 4,296) yuan, and 1,969 (IQR, 1,590) yuan, respectively. The cost of group P was significantly higher than for group M and group M-P (*P* < 0.01), while no differences were observed between group M and group M-P. The total costs of ICU treatment were 81,123 (IQR, 41,311) yuan, 66,941 (IQR, 55,123) yuan, and 57,634 (IQR, 55,474) yuan in the three groups, respectively. The cost of group M-P was distinctly lower than for group M and group P, but the significant difference was detected only between group M and group M-P (*P* < 0.01).

**Table 4:** Pharmacoeconomics of sedation in the ICU

**Data**	**1 Group M (*n*** **= 43)**	**2 Group P (*n*** **= 42)**	**3 Group M-P (*n*** **= 39)**	***P *** **value**	***Post hoc *test (*P*** **< 0.017)**
Pharmaceutical sedation cost (yuan)	1,982 (2,348)	3,744 (4,296)	1,969 (1,590)	<0.01	1 vs. 2; 2 vs. 3
Total cost of treatment in ICU (yuan)	81,123 (41,311)	66,941 (55,123)	57,634 (55,474)	0.04	1 vs. 3

Data expressed as median (interquartile range). Kruskal–Wallis analysis of variance was used for data comparison. In the presence of significant difference in the three groups (*P* < 0.05), the parameters between any two groups were further compared with the value of α adjusted to 0.017.

Data pertaining to the pharmaceutical sedation costs and total ICU cost analysis by treatment received are presented in Table 5. The pharmacy cost of group M-P was significantly lower than for group P (*P* < 0.01) and its total ICU cost was significantly lower than for group M (*P* = 0.015).

**Table 5:** **Pharmacoeconomics of sedation in the ICU**^**a **^**using treatment analysis**

**Data**	**1 Group M (*n*** **= 37)**	**2 Group P (*n*** **= 35)**	**3 Group M-P (*n*** **= 32)**	***P *** **value**	***Post hoc *test (*P*** **< 0.017)**
Pharmaceutical sedation cost (yuan)	1,981 (2,536)	3,538 (4,075)	2,143 (2,032)	< 0.01	1 vs. 2; 2 vs. 3
Total cost of treatment in ICU (yuan)	86,117 (45,269)	6,748 (44,715)	59,756 (49,383)	0.04	1 vs. 3

Data expressed as median (interquartile range). Kruskal–Wallis analysis of variance was used for data comparison. In the presence of significant difference in the three groups (*P* < 0.05), the parameters between any two groups were further compared with the value of α adjusted to 0.017. ^a^Calculation of 104 patients in the per-treatment-received analysis.

### Memories about discomfort to mechanical ventilation-related events

A total of 135 patients were enrolled in the study, 28 patients were lost to follow-up, and 104 patients completed the questionnaire on their recollection of mechanical ventilation-related events. Their combined responses to these events of discomfort during sedation period were analyzed (shown in Table 6). The proportion of unbearable memories involving these events of discomfort in group M-P was lower than the other two groups. Statistically significant difference was only detected between group M and group M-P (*P* < 0.01). The proportion of bearable memories in group M was lower than in the other two groups (*P* < 0.01). The proportion with no memory in group M and group M-P was similar, and higher than in group P. The only significant difference was detected between group M and group P (*P* < 0.01).

**Table 6:** Recall of actual mechanical ventilation-related events (cases)

**Data**	**1 Group M**	**2 Group P**	**3 Group M-P**	***P *** **value**	***Post hoc *test (*P*** **< 0.017)**
Number of total events (cases)	148 (37)	140 (35)	128 (32)		
Unbearable memories	37 (25.0%)	26 (18.6%)	15 (11.7%)	0.02	1 vs. 3
Bearable memories	65 (43.9%)	89 (63.6%)	81 (63.3%)	<0.01	1 vs. 2; 1 vs. 3
No memory	46 (31.1%)	25 (17.9%)	32 (25.0%)	0.03	1 vs. 2

Data expressed as number (percentage). Data comparisons were based on the chi-squared test. In the presence of significant difference in the three groups (*P* < 0.05), the parameters between any two groups were further compared with the value of α adjusted to 0.017.

### Adverse effects

Hypotension occurred in 22 patients (52.4%) of group P, 10 patients (23.3%) of group M and eight patients (20.5%) of group M-P during the sedation period, including 10 episodes of unacceptable hypotension (decrease in systolic blood pressure >40 mmHg or systolic blood pressure <80 mmHg: three episodes in group M, five episodes in group P, and two episodes in group M-P). The incidence of hypotension in group P was significantly higher than in the other two groups (*P* = 0.01). No patients showed hypertriglyceridemia in group P and group M-P. Fifteen patients died during the ICU stay (nine due to multiorgan failure, two due to refractory hemorrhage after operation, and four due to severe acute respiratory distress syndrome).

## Discussion

Our study showed that the sequential use of midazolam and propofol for long-term sedation was an effective and safe sedation protocol. The study showed a less frequent incidence of agitation, faster recovery, earlier extubation and lower total ICU cost, and had a trend to accelerate ICU discharge compared with midazolam alone. The protocol was associated with less cost of pharmaceutical sedation, and less incidence of hypotension compared with propofol alone. No patients showed hypertriglyceridemia with propofol used alone or sequentially. The use of propofol in the long-term sedation was associated with faster recovery, earlier extubation and shorter mechanical ventilation, increased incidence of hypotension and higher pharmaceutical sedation costs than midazolam.

In the present study, no differences were seen in terms of adequate levels of sedation among the three groups. This finding was consistent with the previous published results [23]. The quality of sedation of midazolam and propofol (used alone or sequentially) was similar. Withdrawal symptoms may occur in ICU patients with long-term exposure to benzodiazepine sedatives [24]. Midazolam is a water-soluble benzodiazepine with a rapid onset and a short duration of action, withdrawal syndrome (including agitation) immediately following cessation. The rate of agitation after interruption of sedation in group M-P was significantly lower than group M, similar to the results of Saito and colleagues who found that the rate of agitation evaluated after extubation in group M-P (8%) was significantly lower than in group M (54%) [23]. Although withdrawal syndrome in critically ill patients may be attributed to other causes, such as alcohol or illicit-drug withdrawal, or chronic use of anxiolytics or hypnotics, our study population excluded these patients with addiction. The decreased incidence of agitation in group M-P may be attributed to a period of propofol infusion before stopping sedation, masking withdrawal symptoms associated with discontinuation of midazolam.

Many studies demonstrated that the use of propofol for long-term sedation in the ICU significantly shortened the extubation and recovery times, in comparison with midazolam [6-8,12,13]. In the present study, the average recovery and extubation times and the mechanical ventilation time in group P and group M-P were significantly shorter than in group M (*P* < 0.05). The differences in recovery and extubation times between group P and group M were in accordance with the findings of Hall and colleagues, showing that the extubation time in long-term sedation (more than 3 days) was 46.8 hours in patients sedated with midazolam and 8.4 hours in patients sedated with propofol [6]. The recovery time was 54.7 and 1.8 hours, respectively, for both the groups. While Saito and colleagues reported that the recovery time in group M-P was only 3 hours shorter (average) than group M, the sequential use of midazolam and propofol was not associated with significant advantage [23]. In the study conducted by Saito and colleagues, 13 patients with midazolam were switched to propofol approximately 24 hours before the expected cessation of sedation [23]. When patients were sedated with midazolam for a longer time, extubation delays of up to 49 hours or even longer were reported [13,25]. After interruption of midazolam infusion, the recovery time and extubation time exhibited individual differences [2,6]. The recovery time was longer under signs of extreme agitation, with the larger dosage and more frequent administration of the sedative. Therefore, based on the need for tracheal intubation, respiratory support could probably be synchronized with the requirement for sedation. The switching from midazolam to propofol was based on the weaning process after the patients passed the SBT safety screen. Midazolam was switched to propofol as early as possible in an effort to mask the accumulation of midazolam and also to relatively shorten the duration of midazolam infusion, and then produce more rapid awakening and earlier extubation, and to shorten the duration of mechanical ventilation. Midazolam and fentanyl used in the long term may result in drug accumulation and delayed drug effect, especially in patients with chronic renal failure. No patient with chronic renal failure was present in the three groups. Also, during the study period, no differences existed in the incidence of acute kidney injury and the number of patients receiving continuous renal replacement therapy among the group (*P* > 0.05).

In this study, the ICU duration in group M-P was shorter than in group M (*P* = 0.016) in the per-treatment-received analysis, and using an intention-to-treat analysis the sequential use of midazolam and propofol also had a trend to accelerate ICU discharge compared with midazolam alone (*P* = 0.018). Comparing propofol with midazolam in the long-term sedation, the role of propofol sedation in accelerating ICU discharge is still controversial. The studies conducted by Carrasco and colleagues and Barrientos-Vega and colleagues demonstrated that the use of propofol was associated with earlier ICU discharge [7,13]. However, Sanchez-Izquierdo-Riera and colleagues and Hall and colleagues (using *post hoc* analysis) found that the length of ICU stay was longer for propofol-sedated patients than midazolam-sedated patients [6,11]. Problems persisted following discharge of patients after extubation from the ICU. The patient's disease process still required further ICU care after extubation. Unfortunately a shortage of floor beds was also seen.

In this study, the pharmaceutical cost of sedation and the total ICU cost were considered for the cost–benefit analysis of sedation in the three groups. The pharmaceutical sedation costs in group P were significantly higher than for group M and group M-P. Additionally, the total ICU cost in group M was the highest in the three groups, but the only statistically significant difference was measured between group M-P and group M. Carrasco and colleagues and Weinbroum and colleagues found that the pharmaceutical cost of propofol was higher than midazolam in the long-term sedation [7,14]. Barrientos-Vega and colleagues reported for the long-term sedation that the cost attributed to sedation of midazolam was significantly lower than for propofol and the cost per patient in the propofol group (including ICU therapy and sedation with midazolam) was $1,362 less than in the midazolam group [13]. Although there are remarkable differences in the cost of sedation with these two agents, the economic effect was more favorable for propofol than for midazolam due to a shorter weaning time associated with propofol administration. The sequential use of midazolam and propofol produced the greatest cost–benefit of sedation, compared with propofol, due to the decrease in the pharmaceutical sedation cost. Compared with midazolam, the sequential use decreased the total ICU cost through the decrease in the mechanical ventilation time and length of ICU stay.

Research showed that midazolam produced anterograde amnesia and the amnesic effect was better than for propofol [14]. In this study, the rates of unbearable memories in group M-P was apparently lower than in the other two groups. The proportion with no memory in group M and group M-P was higher than in group P. These results showed that amnesia was evident when midazolam and propofol was sequentially used for sedation in the critically ill mechanically ventilated patients.

A higher number of therapeutic failures because of sedative inefficacy was seen in the propofol group compared with the midazolam group [26]. We also found that two patients had therapeutic failure in group P, due to inadequate sedation at the highest dosage of sedative. Cardiovascular depression was one of the adverse effects with both sedatives. Hypotension was greater with propofol than midazolam during induction [7,8,11,12,14,27]. In this study, the incidence of hypotension in group M-P was significantly lower than in group P, as midazolam was switched to propofol without a loading dose. All of the events were quickly resolved with vasopressor agents or plasma volume expansion [28]. The use of propofol for long-term sedation was associated with hypertriglyceridemia [7,8,11,13]. In the patients sedated with propofol during the this study period, propofol was considered part of the nutrition support, and the patients' calorie intake from propofol infusion was deducted from their parenteral nutrition or enteral nutrition. Hence, none of these patients presented with hypertriglyceridemia. Furthermore, no other serious adverse events were observed in the study.

There are limitations to this study. The study was not blinded, as the physical appearance of the two sedatives was obviously different. A possibility of knowledge bias by the nurses was another limitation. However, almost 200 nurses were randomly involved in the care of all the patients during the ICU stay in our sedation protocols. Hence, the likelihood of bias was negligible. Delirium was not assessed as it was left to the treating physicians.

## Conclusion

Compared with midazolam, the sequential use of midazolam and propofol for long-term sedation reduced the incidence of agitation after the cessation of sedation. This sequential use was associated with a faster recovery, earlier extubation, a shorter mechanical ventilation time and a trend to accelerate ICU discharge as well as decreased total ICU cost. Other advantages included less pharmaceutical sedation cost, and lower incidence of hypotension compared with propofol alone. The sequential use of midazolam and propofol was therefore a safe and effective sedation protocol, with higher clinical effectiveness and better cost–benefit ratio than midazolam or propofol alone in the long-term sedation of critically ill, mechanically ventilated patients. Further research is recommended to elucidate the clinical application of this protocol.

## Key messages

• The sequential use of midazolam and propofol for long-term sedation was an effective and safe sedation protocol.

• Compared with midazolam, the sequential use of midazolam and propofol reduced the incidence of agitation after the cessation of sedation, led a faster recovery, earlier extubation and shorter mechanical ventilation time, and had a trend to accelerate ICU discharge and lowered the total ICU cost.

• The sequential use of midazolam and propofol was associated with less pharmaceutical sedation cost, and a lower incidence of hypotension compared with propofol alone.

• The use of propofol in the long-term sedation was associated with faster recovery, earlier extubation and shorter mechanical ventilation, increased incidence of hypotension and higher pharmaceutical sedation cost than midazolam.

## Abbreviations

IQR: interquartile range; SAS: Riker sedation–agitation scale; SBT: spontaneous breathing trial.

## Competing interests

The authors declare that they have no competing interests.

## Authors’ contributions

YFZ designed and conducted the study, collected and analyzed the data, and wrote and revised the manuscript. YK designed and conducted the study, and wrote and revised the manuscript. XDJ designed and conducted the study, and wrote the manuscript. GPL conducted the study, analyzed the data, and revised the manuscript. TTL designed the study, collected and analyzed the data, and assisted with writing the manuscript. ND collected and analyzed the data, and assisted with writing the manuscript. All authors read and approved the final manuscript.
